# Detection of Genes in *Arabidopsis thaliana* L. Responding to DNA Damage from Radiation and Other Stressors in Spaceflight

**DOI:** 10.3390/genes12060938

**Published:** 2021-06-19

**Authors:** Vidya Manian, Jairo Orozco-Sandoval, Victor Diaz-Martinez

**Affiliations:** 1 Department of Electrical & Computer Engineering, University of Puerto Rico, Mayaguez, PR 00681-9000, USA; jairo.orozco@upr.edu (J.O.-S.); victor.diaz16@upr.edu (V.D.-M.); 2 Bioengineering, University of Puerto Rico, Mayaguez, PR 00681-9000, USA

**Keywords:** ionizing radiation, topological and algebraic spectral network analysis, Pearson correlation, non-radiation-induced genes, Markov blanket, causal relation, logistic regression, Jaccard similarity index, flavonoids and carotenoids

## Abstract

Ionizing radiation present in extraterrestrial environment is an important factor that affects plants grown in spaceflight. Pearson correlation-based gene regulatory network inferencing from transcriptional responses of the plant *Arabidopsis thaliana* L. grown in real and simulated spaceflight conditions acquired by GeneLab, followed by topological and spectral analysis of the networks is performed. Gene regulatory subnetworks are extracted for DNA damage response processes. Analysis of radiation-induced ATR/ATM protein–protein interactions in *Arabidopsis* reveals interaction profile similarities under low radiation doses suggesting novel mechanisms of DNA damage response involving non-radiation-induced genes regulating other stress responses in spaceflight. The Jaccard similarity index shows that the genes AT2G31320, AT4G21070, AT2G46610, and AT3G27060 perform similar functions under low doses of radiation. The incremental association Markov blanket method reveals non-radiation-induced genes linking DNA damage response to root growth and plant development. Eighteen radiation-induced genes and sixteen non-radiation-induced gene players have been identified from the ATR/ATM protein interaction complexes involved in heat, salt, water, osmotic stress responses, and plant organogenesis. Network analysis and logistic regression ranking detected AT3G27060, AT1G07500, AT5G66140, and AT3G21280 as key gene players involved in DNA repair processes. High atomic weight, high energy, and gamma photon radiation result in higher intensity of DNA damage response in the plant resulting in elevated values for several network measures such as spectral gap and girth. Nineteen flavonoid and carotenoid pigment activations involved in pigment biosynthesis processes are identified in low radiation dose total light spaceflight environment but are not found to have significant regulations under very high radiation dose environment.

## 1. Introduction

Space is an environment that may have harmful effects on living organisms due to its hostile characteristics such as low oxygen, microgravity, lower pressure, and radiation. Radiation is the emission of energy as electromagnetic waves or as moving subatomic particles, especially high-energy particles. Radiation is of two types: non-ionizing and Ionizing Radiation (IR). The radiation experienced in low Earth orbit is primarily low Linear Energy Transfer (LET) present in the International Space Station (ISS). Organisms in space are exposed to radiation, which affects the health of humans and the anatomy and growth of plants and animals [1]. Space missions have to deal with IR in current spacecraft and future long-term deep-space missions [2]. Plants are affected by radiation resulting in damage to cellular components and damage to Deoxyribonucleic Acid (DNA) [3]. Exposure to radiation causes DNA lesions in plant cells such as single-stranded breaks, Double Strand Breaks (DSB), mismatches, and modified bases. This leads to DNA Damage Response (DDR) that includes signal transduction, triggering either DNA repair, cell survival, or cell death [4]. 

The plant *Arabidopsis thaliana* (*Arabidopsis*) is a member of the mustard family widely used as a model organism in plant biology for studies of the cellular and molecular biology of flowering plants. It has a rapid life cycle of about six weeks from germination to mature plants. It can be easily cultivated in restricted space, has a small genome, a large number of mutant lines, extensive genetic maps, and genomic resources. Hence, it has been chosen as a model organism for spaceflight studies by research communities [5,6]. Similar to observations made on terrestrial plant stressors, spaceflight missions have made extensive observations and collected transcriptional gene regulatory datasets of *Arabidopsis* [7]. Plants have evolved genes that participate in DNA repair pathways that help the plant to survive the effects of radiation. Exposure to IR in *Arabidopsis* has shown a decrease in DNA methylation with no effect on morphology or root growth or seed germination [8]. Ataxia-telangiectasia and Rad3-related protein (ATR) and Ataxia-Telangiectasia Mutate (ATM) are *Arabidopsis* homologs of the human ATR and ATM genes, which are activated by DNA single and double-stranded breaks in eukaryotes [9]. ATM-dependent transcriptional changes in *Arabidopsis* due to IR and its influence on root growth are discussed in [10]. Two mutants of *Arabidopsis* defective in the ATR and ATM protein kinases have shown hypersensitivity to radiation resulting in the upregulation of hundreds of genes, while both ATR and ATM mutants responded with transcriptional changes to DNA damage due to radiation [11]. The effect of acute radiation on *Arabidopsis* growth and its impact on protein-coding genes is presented in [12]. 

Apart from DDR, plants adopt pigment biosynthesis processes to protect them against Ultraviolet (UV) radiation. These pigments are flavonoids, betalains, and carotenoids. Flavonoids provide a range of color from pale-yellow to blue and belong to the class of phenylpropanoids [13]. Carotenoids contain up to 15 conjugated double bonds [14]. It has been shown that light induces expression of carotenoid genes during leaf, flower development, and fruit ripening in *Arabidopsis* [15]. Carotenoids protect the plant against excessive light and add color to the flowers, fruits, and seeds [16].

163 ATR/ATM genes in *Arabidopsis* have been shown experimentally as responding to ionizing radiation in [11]. We call these ATR/ATM genes radiation-induced genes and other downstream genes of stress signaling non-radiation-induced genes [17]. There is no putative radiation subnetwork analysis for detecting radiation and non-radiation induced genes that are involved in biological processes related to radiation response in the plant. In this paper, we are analyzing the effects of low LET radiation on *Arabidopsis* found in low Earth orbit experienced by current spaceflight missions STS-129 and SpaceX. As long term spaceflight missions beyond the shielding effects of Earth’s atmosphere and magnetic field are contemplated, we also analyze the effects of two different types of IR on *Arabidopsis*: gamma photons, which have low rates of linear energy transfer (LET), and relativistic Fe nuclei (here termed HZE), a high LET form of IR [18]. The regulation of the Carotenoid Biosynthesis Process (CBP) at the transcriptional level is important for the production of plant hormones, photosynthesis, and dispensing of seeds. In this paper, we investigate CBP regulation in the spaceflight environment, as well.

As modules are more informative for the regulatory mechanism of a biological process [19], we perform module-based analysis and have identified radiation and non-radiation induced gene/protein interactions in DNA repair processes, as well as flavonoids and carotenoids that respond to light environment in spaceflight. We call these modules subnetworks. The *hypothesis* of this research is: if subnetworks of radiation-induced biological processes are extracted, then hub genes and target (authority) genes including non-radiation induced genes can be identified, because network analysis detects and ranks hub genes and authority genes. Furthermore, topological and algebraic spectral network analysis enables the comparison of the subnetworks for different doses of radiation, revealing similarities and differences in the regulation of these processes. 

## 2. Materials and Methods

Datasets from the GeneLab repository [20] are mined for studying the effects of low radiation doses in low Earth orbit, and very high doses of high LET and gamma photos found beyond Earth’s atmosphere on *Arabidopsis*. All the omics datasets in GeneLab are preprocessed and normalized before being published. The radiation metadata for GLDS-7, 120, 37, 38, and 46 are given in Supplementary Table S1.

### 2.1. GeneLab Datasets

GLDS-7: the dataset contains microarray gene expression data from the tissues (roots, leaves, and hypocotyls) of the plant *Arabidopsis Wassilewskija* (WS) and *Columbia* (Col-0) ecotypes grown in spaceflight and ground control [21]. Ground control experiments were conducted using identical ABRS hardware and an environmental chamber programmed to International Space Station (ISS) environmental conditions. The data contains 20,000 genes with five expression values per component.

GLDS-120 dataset contains the transcriptomics data of the *Arabidopsis* WS and Col-0 ecotypes, and the gene *Phytochrome D* (*phyD*) mutant of ecotype Col-0. The different stressors imposed on the plants are light and dark in spaceflight, and light and dark on the ground. Gene expression data are available for pairs of combinations of genotypes and ecotypes [22,23].

The GLDS-7 and GLS-120 datasets were collected under a low dose of radiation, with an average absorbance of 1.25 milliGray (mGY) and an average absorbance of 1.655 mGY for low-LET (SAA), and an average cumulative absorbed dose of 1.86 mGY. 

GLDS-37 dataset contains the transcriptomic data of four different ecotypes of *Arabidopsis* in spaceflight microgravity and on the ground: Col-0, LER-2, WS-2, and Cvi-0. The seeds of the mentioned ecotypes are germinated in orbit and grown for eight days [24]. Ground control data is collected under the same environmental stressors. RNAseq is performed to analyze the transcriptomic changes of the ecotypes.

GLDS-38 dataset contains proteomics and transcriptomics data of the *Arabidopsis* seedlings. The seeds were flown in the International Space Station (ISS) and allowed to germinate and grow for three days in microgravity [25]. *Arabidopsis* Wild Type Col-0 seeds were plated onto twenty-two 60 mm Petri plates, loaded into PDFUs, and inserted 4 Biological Research in Canisters (BRICs).

The GLDS 37 dataset was collected under a low radiation dose with a cumulative absorbed dose of 1.06 mGY. The GLDS-38 data was collected under a low radiation dose with a cumulative absorbed dose of 0.38 mGY. The above four datasets correspond to radiation exposure in low Earth orbit spaceflights.

GLDS-46 dataset was collected under HZE (1 Giga electron Volt (GeV) Fe^26+^ high mass, high charge, and high energy relativistic particles) and photons (γR) which have low rates of Linear Energy Transfer (LET) on *Arabidopsis* seedlings. Both types of radiation showed DSB damage response. This dataset was collected under a very high dose of 100 Gy at a dose rate of 1.8 Gy/min. A 100 Gy radiation dose in *Arabidopsis* would generate the same number of DSBs as a 4Gy dose in humans. We are interested in this dataset because of foreseen long-term manned missions beyond the Earth’s atmosphere and magnetic field to the moon and Mars [18]. 

### 2.2. Gene Regulatory Network Inferencing Using Pearson Correlation

We define a graph-based representation for the gene expressions. Formally, a graph is a pair of sets G:= (V,E) where |V| is the set of vertices (molecules, genes, proteins, nodes, points) and |E| is the set of edges, which is an ordered pair of V. The graph (V, E, o, t) is called directed, if directed edges are allowed, i.e., not all edges have reverse edges as members of E. In a directed graph G = (V,E,o,t), the edges e = (u,v), the origin of e is denoted by o and the terminal v is denoted by t(v).

The Pearson’s correlation coefficient between two variables (genes, nodes) is defined as the covariance of the two variables (x and y) divided by the product of their standard deviations. Gene expression values ranging from three to eight for each gene are used to compute Pearson correlation. The expression values are linearly related, hence Pearson correlation is used [26]. We have applied Pearson correlation network analysis to identify key gene players for cell wall biosynthesis, and root growth in *Arabidopsis* in spaceflight using the gene expression values from GLDS-7 and GLDS-120 datasets [27]. The Pearson correlation coefficient is used as the measure of correlation (between −1 to 1) and gene pairs are considered co-expressed if their *p*-value is less than or equal to the corrected threshold *p*-value. The *t*-test is used to establish if the correlation coefficient is significantly different from zero, and, hence there is evidence of an association between the two variables. The genes are treated as nodes and the relation between them is an edge. Therefore, the genes with the highest degree are related to a higher number of target or authority genes. Those genes that have the highest degree or intramodule (within graph) connectivity are treated as hub genes to construct the gene regulatory networks (GRNs). Vertices (u,v) are the edges (arcs), respectively [28]. A graph is weighted if each edge e is assigned a real-valued weight w. In our approach, the weights of the edges between two nodes (genes) are the correlation values.

### 2.3. Causal Relations Discovery Using Incremental Association Markov Blanket (IAMB) Method

Causal relations are difficult to infer and require careful application of experimental interventions. However, causal relations can be discovered by statistical analysis of purely observational data, which is known as causal structure learning [29]. Using Markov properties, a gene is conditionally independent of all other genes except its parents, children, and children’s parent variables (genes). Causal relationships are useful for combining omics data with Genome Wide Association Studies (GWAS), for inferring relationships between genotype and phenotype [30]. 

The method used for causal relation inferencing here is the Markov Blankets (MB) method and Bayesian Network (BN) learning [31,32,33,34]. Joint conditional probabilities are represented by a graph in a Bayesian network, the nodes (genes) are connected by the Markov property which states that a node is conditionally independent of its nondescendants, given its parents. Applying the faithfulness condition, the IAMB of any node (gene) in a BN is the set of parents, children, and spouses (the other parents of their common children) of the gene [32]. We determine the direct causes of a gene’s upregulation, the effects on a gene or a protein due to the upregulation of the gene, as well as the upregulation of the causes of the activation of the gene. In our case, each gene is a variable with a series of expression values. The Markov blanket of a gene X is the smallest set MB(X) containing all genes carrying information about X that cannot be obtained from any other gene. The MB(X) of a node (gene) X includes its parents, children, and spouses which are the strongly relevant genes to gene X; that is, of genes that support and are associated with the target gene (X). Association measures and conditional independent tests are applied to identify the strongly relevant genes [35,36]. Hence, MB(T) is a causal structure learning algorithm useful for the discovery of regulatory interactions among genes from gene expression data.

### 2.4. Computation of Network Measures

The network measures include spectral gap, girth or diameter, density, connected components, and Jaccard similarity [28,37,38]. These are used to compare the subnetworks for each of the processes.

Spectral gap: For a graph G, the Laplacian eigenvalues can be ordered as 1 = |λ_1_| ≥ |λ_2_|≥ · · · ≥|λn| (G may be directed or undirected, weighted or unweighted, simple or not). The Spectral gap is defined as: δ_λ_ = |λ_1_| − |λ_2_|. By normalizing the Laplacian matrix of G, the eigenvalues are λ_1_ ≥ λ_2_ ≥ · · · ≥ λn > 0, and the Laplacian spectral gap will be: δ_λ_ = 1 − |λ_2_|. The spectral gap is also known as a random walk, in terms of this concept λ_2_ is the most important eigenvalue. Note that if the spectral gap is 0, which means λ_2_ = 1 (Γ is not (strongly) connected or if Γ is bipartite), this means a typical random walk will not converge to a unique distribution or dominant eigenvector. As long as the spectral gap is greater than 0, which means |λ_2_| < 1, then the random walk converges to a unique dominant eigenvector, and the spectral gap measures the rate of convergence, the larger the spectral gap (the smaller|λ_2_|), the better the network flow (large h(G), diffusion, mixing, random walk, expansion, sparsity, and other highly desirable properties of the network G). 

Girth of a graph is the smallest positive integer r such that Trace(A^r^) > 0. Let d = d(G) be the smallest integer (if it exists) so that for every pair of vertices (u,v) there is a walk of length at most d from u to v. Then d(G) is called the *diameter* or maximum eccentricity of the graph G. 

Density of a graph is the ratio between the number of edges and the number of possible edges. Density is a measure of the compactness of a module (subnetwork) and measures the connectivity strength of pairs of genes in the module [19]. 

Connected component in a directed graph is a subgraph in which any two vertices are connected by paths, and which is connected to no additional vertices in the super graph.

Jaccard similarity index is used to quantify the neighborhood similarity between two vertices (genes), it is also called topological similarity [39]. The Jaccard index calculates the proportion of v-type genes between two u-type genes, relative to the total number of v-type genes connected to either u-type genes [40]. An index of 1 indicates the exact similarity between u and v, while 0 indicates no similarity between the two sets. The above measures are computed for the subnetworks. If the Jaccard similarity is 0.5, it indicates that half of the genes in one subnetwork are also found in the other subnetwork. Furthermore, hub genes of subnetworks are compared using the degree distribution and subgraph centrality measures.

Degree distribution is the number of neighbors connected to a node; in other words, it is the number of edges incident on a node. The degree distribution can give information about the structure of a network. The networks can be directed or undirected. In the undirected case, the degree of node *i* is the number of connections it has, and it can be represented as an adjacency matrix, with the sum over all nodes. For directed graphs, there are two types of degree distributions: in-degree, which is the number of connections entering the node, and out-degree, which is the number of outgoing connections. The degree distribution for a directed graph is the fraction of vertices of degree k_in_ and k_out_. In this case, the degree distribution is computed for the hub genes in the four low radiation dose subnetworks.

Subgraph Centrality of a node is a weighted sum of closed walks of different lengths in the network starting and ending at a node. Centrality measures are used widely in biological networks to infer protein-protein interactions and identify essential proteins [41,42,43].

### 2.5. Logistic Regression-Based Gene Ranking

Logistic regression, also called a logit model, is a statistical method for analyzing a dataset in which there is any number of variables that determine an outcome. The goal of this method is to find the best fitting model to describe the relationship between a dichotomous characteristic of interest (dependent variable; response or outcome variable) and the explanatory variable. It is used to model the log-odds of a gene belonging to a specific category as a linear function of the statistical significance. Most likely enriched gene sets will be identified based on the *p*-value or based on the odds ratio if a ranking independent of category size is desired [44]. The logistic regression method is an extension of the χ2-test and has higher statistical power than other methods because the important values do not depend on a threshold.

**Figure 1:**
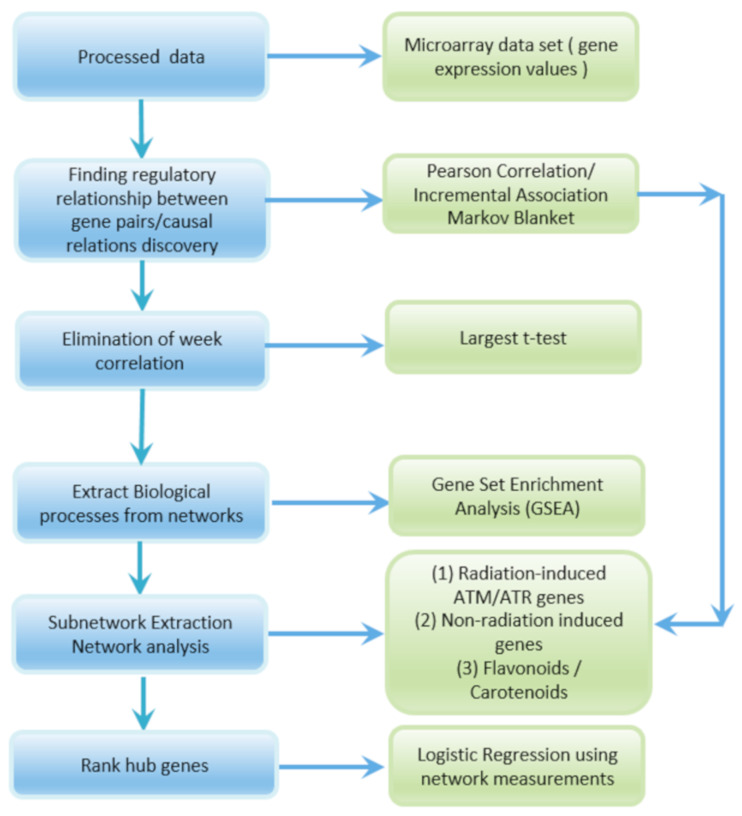
Flow diagram showing the sequence of steps followed for extraction of radiation response subnetworks from the gene expression values in the GLDS datasets and network analysis.

Figure 1 shows the steps involved in the extraction of radiation subnetworks. Pearson correlation and IAMB methods outlined in Section 2B and 2C are applied to the GLDS-7, 120, 37, 38, and 46 expression values of *Arabidopsis* Col-0 ecotype in spaceflight to construct the GRNs. Weak correlations are eliminated by thresholding *p*-values which correspond to the largest *t*-test statistics in the Pearson correlation GRNs. GSEA is used to extract subnetworks of significant DDR processes with a majority of higher activations of radiation-induced ATR/ATM genes. Radiation-induced ATR/ATM hub genes are identified in the subnetworks. In these subnetworks, genes that have strong correlations with radiation-induced ATR/ATM genes are detected. The genes significantly altered due to other spaceflight parameters that are not correlated with ATR/ATM genes are not detected. Network measurements listed in Section 2D are computed and used to analyze the subnetworks. Certain non-radiation-induced genes that interact with the radiation-induced hub genes are detected in the DDR processes subnetworks. The associated processes and molecular functions of the key non-radiation induced genes that interact with the ATR/ATM radiation-induced hub genes are identified. Flavonoid and carotenoid biosynthesis processes are identified from the Pearson GRNs and MB causal relational networks and their corresponding subnetworks are extracted. The carotenoid and flavonoid hub genes are detected in the subnetworks. The logistic regression method explained in Section 2E takes the network measurements for the radiation-induced hub genes in the subnetworks as input and ranks them from the most to the least significant.

The Pearson correlation GRN construction is implemented in Python, and the causal relational analysis using the IAMB method is implemented in R. The computational algorithms for the network measures, and logistic regression ranking of hub genes are implemented in Python. The github link for the scripts is provided in the Supplementary Material.

## 3. Results

The gene expression values (three in GLDS-120, three in GLDS-38, five in GLDS -7, six in GLDS-37, and eight in GLDS-46) are used to construct Pearson correlation GRNs. The datasets are analyzed separately. Weak correlations with *p*-values greater than 0.0005 are removed. A Gene Set Enrichment Analysis (GSEA) [45] of the Pearson correlation GRNs for low radiation dose datasets reveals four main processes with higher activation of the ATR and ATM genes. They are cellular response to stimulus, cellular response to stress, cellular response to DNA damage stimulus, and DNA metabolic processes. The radiation protection pigment biosynthesis processes identified are the flavonoid biosynthesis process and carotene catabolic process. The subnetworks for these processes are extracted from the GRNs for each dataset. The hub genes are detected by thresholding the degree distribution of the subnetworks. A threshold between 5 and 34 for the degree distribution is used to select the hub genes for the Low Radiation Dose (LRD - LRD1 refers to GLDS-37, LRD2 refers to GLDS-38, LRD3 refers to GLDS-7 and LRD4 refers to GLDS-120) subnetworks. A threshold of 19 to 47 is used to select the hub genes for the very high radiation dose subnetworks (HZE and γR radiation GLDS-46 dataset). The protein-protein interactions in the subnetworks are discussed below. The hub genes for each process are given by their *Arabidopsis* Gene Identifier and their gene names inside the parenthesis. The gene names are also listed in the Supplementary Tables S2 and S3. The gene ontologies of the ATG genes are obtained from [17,46]. 

### 3.1. Cellular Response to Stimulus

Cellular response to stimulus process is the mechanism by which a cell detects and responds to external or internal signaling stimuli causing the cell to change in state or activity in terms of movement, secretion, enzyme production, and gene expression [47]. Table 1 and Table 2 show the degree distribution and subgraph centrality measure for the hub genes in the subnetworks for the cellular response to stimulus process in *Arabidopsis* grown in low radiation dose datasets LRD1 and LRD2, and datasets LRD3 and LRD4, respectively. The higher centrality values in LRD4 subnetwork show the greater occurrence of the three genes AT3G27060 (TSO2), AT3G51920 (CML9), and AT1G70940 (PIN3) (Table 2) in the pathways of other genes and play an active role in the cellular response to stimulus process. A higher value of degree distribution for the hub-gene indicates that it is the most influential gene in the biological pathway. The low radiation dose subnetworks for LRD1 and LRD2 datasets have more hub genes for this process (Table 1). The genes AT3G13380 (BRL3), AT3G61630 (CRF6), and AT5G61600 (ERF104) have higher values than other hub genes in the low radiation dose subnetworks. They are brassinosteroid receptor proteins and ethylene-responsive transcription factors involved in protein and DNA-binding molecular functions.

### 3.2. Cellular Response to Stress

Environmental stressors such as extreme temperature, exposure to toxins, radiation, and mechanical damage result in molecular changes that cells undergo in response to these stressors [48]. Table 3 shows the degree distribution and subgraph centrality measure for the hub genes in the subnetworks for the cellular response to stress process for low radiation dose (datasets LRD1 and LRD2).

The AT5G48720 (XRI1) has a higher degree distribution and subgraph centrality measures in the low radiation dose (LRD2) subnetwork (Table 3). While there are four hub genes involved in cellular response to stress in the low radiation dose LRD1 and LRD2 subnetworks, there is only one hub gene in the low radiation dose LRD3 and LRD4 subnetworks (Table 4). 

### 3.3. Cellular Response to DNA Damage Stimulus 

DNA damage due to environmental stress such as radiation or errors in metabolism causes the cell to change in state or activity in terms of movement, secretion, enzyme production, and gene expression. There are no common hub genes for this process in the LRD1 and LRD2 subnetworks. However, the same gene AT3G27060 that responds to stress also shows higher activity in response to DNA damage stimuli in LRD3 and LRD4 subnetworks. The network measures for this process are given in the second row in Table 4. 

### 3.4. DNA Metabolic Process 

DNA metabolism includes both DNA synthesis and degradation reactions involved in DNA replication and repair [49]. Through this process, the cellular DNA levels are maintained. The LRD1 and LRD2 subnetworks do not have any common hub genes for this process, but the AT3G27060 is involved in this process in the LRD3 and LRD4 subnetworks. It also has high subgraph centrality values in both the cellular response to DNA damage and DNA metabolic processes (second and third rows of Table 4) showing that it is involved in several pathways for both the cellular response to DNA damage and DNA metabolic processes. 

**Figure 2:**
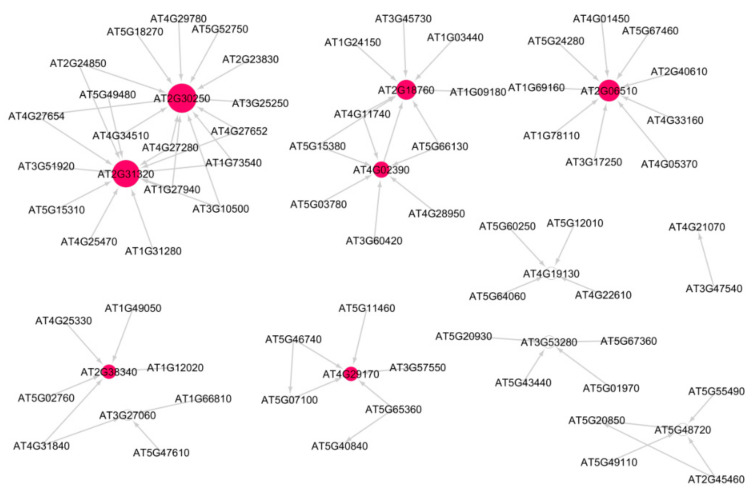
Subnetwork for cellular response to stress process in *Arabidopsis* under low radiation dose (LRD3) dataset. Red circles are hub genes. There are 7 significant hub genes in this subnetwork. Larger the circles, higher the value of in-degree distribution.

**Figure 3:**
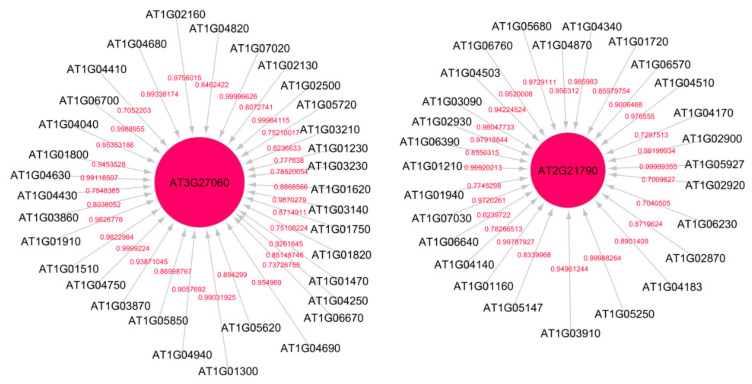
Subnetwork for DNA metabolic process under low radiation dose (LRD4) dataset. Red circles are hub genes. The hub genes AT3G27060 (TS02) and AT2G21790 (RNR1) are being activated by the genes at the non-arrow end of the edge. Both of these genes have a high in-degree distribution.

Figure 2 shows the subnetwork for cellular response to stress process under low radiation dose (LRD3 dataset), and Figure 3 shows the subnetwork for the DNA metabolic process under low radiation dose (LRD4 dataset), respectively. The degree distribution and subgraph centrality have the highest values for the hub genes in the total light low radiation dose subnetwork from the LRD4 dataset as can be seen in Figure 3. All the genes except the hub genes (red circles) are non-radiation-induced in this subnetwork. These genes are involved in responses to osmotic and water stress, and response to cadmium ions, metal ions, inorganic substances, and water deprivation.

### 3.5. Flavonoid Biosynthesis and Carotenoid Catabolic Processes

**Figure 4:**
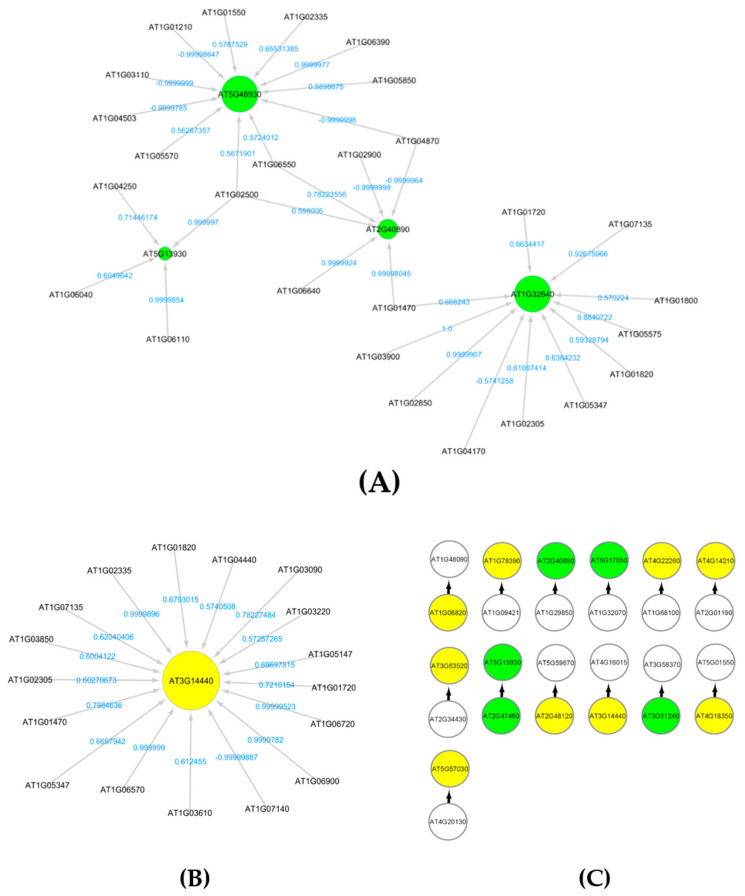
(**A**) Subnetwork for flavonoids biosynthesis process under light environment in space Figure 4. (GLDS-120) dataset. (**B**) Subnetwork showing carotenoid AT3G14440 interactions. This is a key enzyme in the biosynthesis of abscisic acid. It is regulated in response to drought and salinity and expressed in roots, flowers and seeds. (**C**) Markov blanket subnetwork showing causal relations of flavonoids (green) and carotenoids (yellow).

The subnetworks for flavonoid biosynthesis and carotenoid catabolic processes with higher activations of genes that encode and regulate flavonoids and carotenoids are extracted from GRNs of *Arabidopsis* grown in total light environment (LRD4) dataset. Figure 4A,B show the interaction profiles of these genes with green circles for flavonoid encoding genes, and yellow circles for carotenoid encoding genes, respectively. These pigments are involved in UV radiation protection. They have been reported to have higher regulations in several ground-based studies on UV response of *Arabidopsis* [50]. There are higher regulations of flavonoids and carotenoids, which also include negative regulations as indicated by the weights on the edges of Figure 4A,B. From the low radiation dose dataset LRD4, we also find causal relations of five flavonoid genes and nine carotenoid genes, as shown in Figure 4C.

### 3.6. Subnetwork Measurments for the Low, and Very High Radiation Dose DDR Processes 

**Figure 5:**
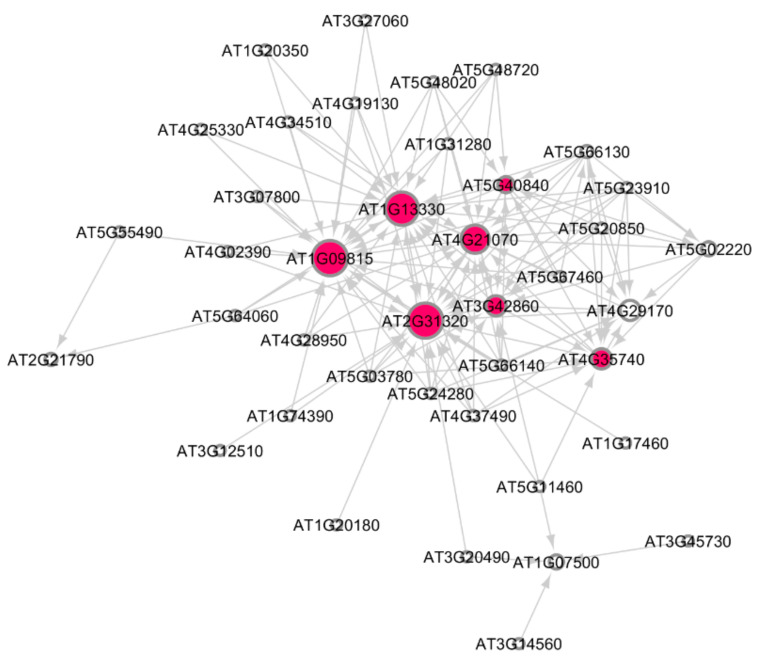
Subnetwork for DNA metabolic process in *Arabidopsis* under very high HZE radiation dose. Hub genes are indicated by red circles. There are seven significant hub genes in this subnetwork. Larger the circles, higher the activation of the hub genes.

**Figure 6:**
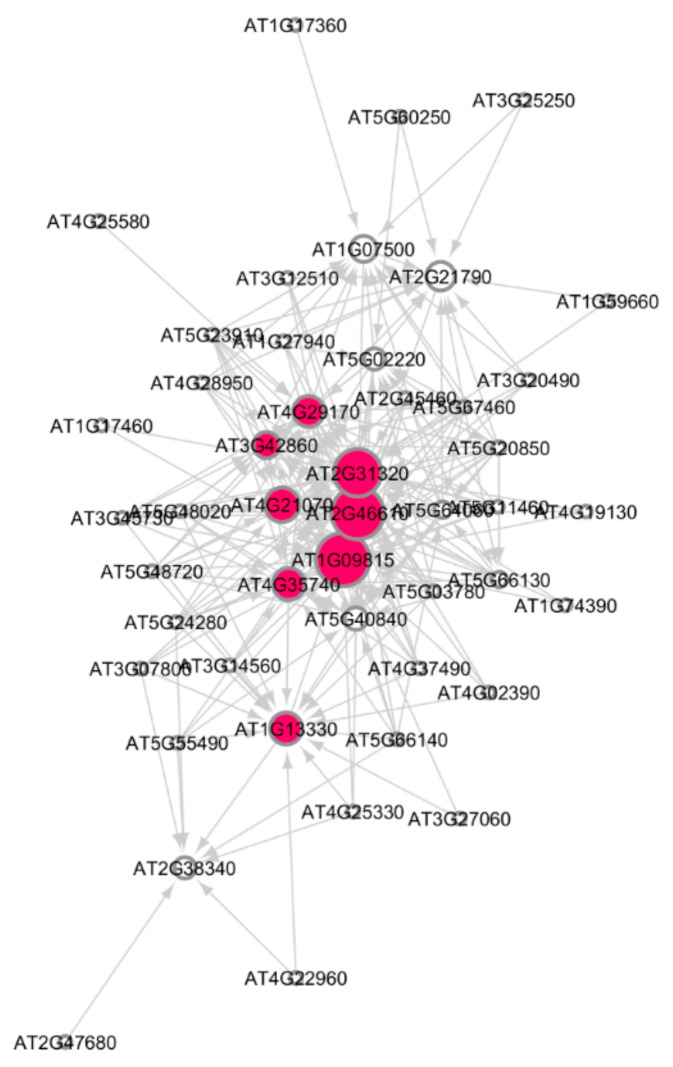
Subnetwork for the nucleic acid response process in *Arabidopsis* under very high γR radiation. Red circles are hub genes. There are nine significant hub genes in this subnetwork. Larger the circles, higher the activation of the hub genes.

We compare the subnetworks for low radiation doses using topological and algebraic network measures. Table 5 summarizes these measures. We constructed GRNs and applied GSEA to extract the subnetworks for DDR processes with a larger regulation of the ATR/ATM genes for the very high radiation dose of HZE and γR radiation dataset (GLDS-46). We analyzed the subnetwork measurements for this dataset to understand the effects of very high doses of radiation on plants grown for life support in long-term deep-space missions beyond the Earth’s atmosphere to the Moon and Mars [18]. The two main processes identified from the GRNs of the very high radiation dose datasets are DNA metabolic process and nucleic acid response process. Figure 5 shows the subnetwork for the DNA metabolic process in *Arabidopsis* under a very high HZE radiation dose. Figure 6 shows the subnetwork for the nucleic acid response process under a very high γR radiation dose. The network measures for these two processes are summarized in Table 6.

The HZE and γR dataset corresponds to a very high radiation dose of 100 Gy at a dose rate of 1.8 Gy/min., and 30 Gy at a dose rate of 2.5 Gy/min., respectively. Hence, the spectral gap measures are much higher for these subnetworks ranging from 7.5153 to 12.2554 as seen in Table 6, whereas the spectral gaps for the low radiation dose subnetworks in Table 5 have lesser values ranging from 0.068 to 0.858. The larger the spectral gap (the smaller |λ_2_|), the higher the network flow with sparseness, expansion, diffusion, and random walk. Hence, these networks have a higher measure of random walks, implying that the nodes that lie closer to each other in the network perform similar functions [51]. This also indicates that the plant has higher transcriptional costs in adapting to the very high levels of radiation in the environment. The low radiation dose subnetworks (LRD3 and LRD4) have a large number of connected components ranging from 3 to 9, while the HZE and γR subnetworks have only one connected component. Also, the low radiation dose subnetworks have an average shortest path of 1.5 and a diameter of 1 or 2. The processes for the very high dose of HZE and γR radiation subnetworks have an average shortest path of 3.25 and a diameter between 2 and 5. Also, there are no non-radiation-induced genes identified in the very high radiation dose subnetworks.

### 3.7. Jaccard Similarity Between Subnetworks

The Jaccard similarity indices in Table 7 show the neighborhood similarity of radiation-induced ATR/ATM hub-genes for the DDR processes influenced by very high doses of HZE and γR radiation. It also shows the similarity of the neighborhood for the hub gene in the two subnetworks for the low radiation dose DDR processes. The values closer to one indicate that the genes AT2G31320 (PARP1), AT4G21070 (BRCA1), and AT2G46610 (RS31A), and AT3G27060 (TSO2) have similar interactions in the subnetworks for the two processes. The closer the values are to zero, the genes have minimal interaction similarity in the neighborhood for the two processes. 

### 3.8. Logistic Regression Ranking of Hub Genes 

The subgraph centrality, closeness centrality, degree distribution, page rank, and eigenvalue centrality network measures are used by the logistic regression method to rank the top correlated genes from the Pearson GRNs. Apart from the hub genes for the subnetworks, the other top-ranked radiation-induced genes from the low radiation dose subnetworks are AT3G21280 (UBP7), AT1G07500 (SMR5), and AT5G66140 (PAD2). Logistic regression of the top correlated genes in the HZE and γR subnetworks resulted in all of the high ranked genes being radiation-induced ATR/ATM genes. No non-radiation-induced genes are among the top 50 ranked genes for the very high radiation dose subnetworks. 

### 3.9. Causal Relational Network Analysis

**Figure 7:**
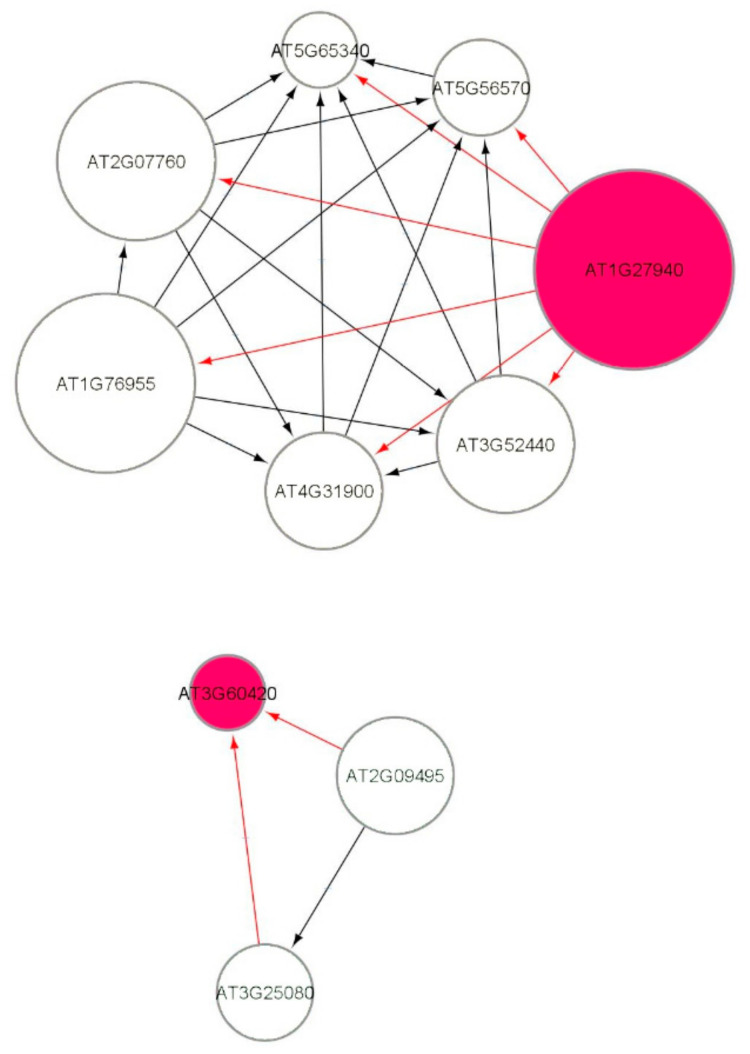
Markov blanket subnetworks for low radiation dose dataset (LRD1).

**Figure 8:**
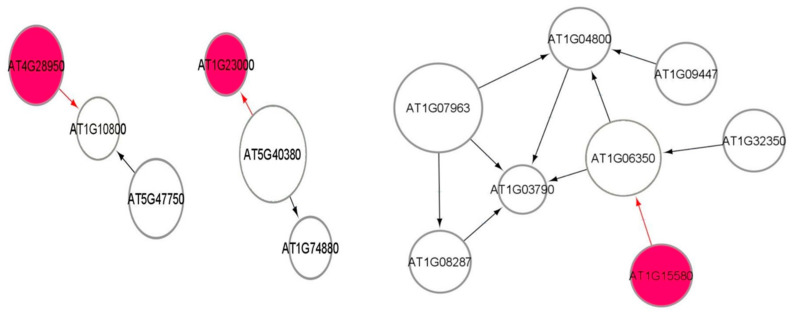
Markov blanket subnetworks for low radiation dose dataset (LRD3—left and LRD4—right).

Figure 7 shows a part of the causal relational subnetwork for the LRD1 dataset. There are two key radiation-induced genes AT1G27940 (Q9C7F8) and AT3G60420 (Q9M217) related to eight non-radiation-induced genes. In a causal graph, for any node (gene) X, its parents and children are the immediate nodes (genes) that are strongly relevant in its activation [36]. The higher activation of a parent gene will lead to the activation of the child gene, as well. The activation of the parent gene is the cause, and the activation of the child gene is the effect. The MB(AT1G27940) has six children genes, and the MB(AT3G60420) has two parent genes, which indicates that this gene has a causal relationship with these two other genes. In addition, there are 82, 72, 69, and 69 one-to-one causal relations between radiation-induced genes and non-radiation genes in each of the LRD1, LRD2, LRD3, and LRD4 subnetworks, respectively. The parent gene carries information most relevant to the child gene, that cannot be obtained from another gene. The key radiation-induced genes for low radiation dose are shown in Figure 8 in red color. The MB(AT4G28950) has one child AT1G10800, MB(AT1G23000) has one parent AT5G40380, and MB(AT1G15580) has one child AT1G06360. The genes that are related to AT3G60420 are involved in plant growth and developmental processes. The genes AT1G10800, AT1G06350 (ADS4), and AT5G40380 (CRK42) (Figure 8) encode proteins and are involved in other biological processes such as ATP binding, protein phosphorylation, fatty acid biosynthesis, and metabolic processes. 

## 4. Discussion

### 4.1. Computational Strategy

Radiation data for GLDS studies in the ISS are provided in [52]. Previous studies of *Arabidopsis* in spaceflight missions have focused on non-radiation stress responses that are due to microgravity, although radiation is an important stressor in spaceflight [47]. The effect of ionizing radiation has been presented using traditional fold change analysis methods in [11,18]. The novelty of our approach is the performance of computational modular subnetwork analysis of biological processes involving a majority of radiation-induced genes. Our modular analysis approach provides a better insight into the complex gene interaction profiles between radiation-induced and non-radiation-induced genes in DDR processes. We have also determined gene associations using the Bayesian Markov blanket method which identifies key genes based on the relevance of gene associations. This method is attractive because of its ability to describe complex stochastic processes with noisy observations. We have used IAMB innovatively in identifying causal relations between ATR/ATM radiation-induced genes and non-radiation-induced genes. We have innovatively used the Jaccard similarity index to show the similarity of neighborhood interactions of ATR/ATM genes in the subnetworks for DDR processes. This index is not biased by low-level, noise-derived, variability in expression [53]. We have also shown how topological network measures of degree distribution are useful for the extraction of subnetwork modules from larger GRNs. The subnetworks sampled from the original GRNs also have a scale-free topology with a degree distribution that follows an exponential power law [54]. Centrality measures are useful for distinguishing protein complexes [55]. It has been shown that the subgraph centrality has better discriminatory power than other centrality measures such as Eigenvector, closeness, and betweenness centrality [56]. Subgraph centrality has been used here to distinguish the hub genes involved in the DDR processes. The network measures of diameter, connected components, and density have important biological implications as they can predict gene regulatory interaction landscape intrinsic to the organism [57]. The density is the lowest for the LRD2 dataset indicating the sparseness of the subnetworks [58]. The network measures for the subnetworks reveal quantitatively different emerging properties for *Arabidopsis* grown in different radiation dose environments. The number of connected components and diameter of the subnetworks for the DDR processes show large variability for low and very high radiation doses. The spectral gap has large values in the very high radiation dose subnetworks than in low radiation dose subnetworks indicating sparseness and high connectivity of the networks. Many biological systems are modular which results from the sparseness in the GRNs [59]. The novel algebraic analysis using spectral gap computations informs us about the diffusion, mixing, and expansion properties of important genes in different radiation dose environments. Through spectral analysis, we have shown that very high doses of radiation, results in large complex biomolecular networks with higher values of the spectral gap. Several machine learning methods such as logistic regression, support vector machine, random forest regression, naïve Bayes classifier, stochastic gradient boosting, and artificial neural networks have been applied to gene expression data to identify differentially expressed genes [60]. The advantage of logistic regression is that it allows the evaluation of multiple explanatory variables. It is easy to implement, interpret, and train. It is better than linear regression which produces continuous output, while logistic regression produces discrete output. This method has ranked the ATR/ATM hub genes of low radiation dose subnetworks, and 50 ATR/ATM genes as the top gene players responding to DNA damage and/or ionizing radiation in spaceflight. 

### 4.2. ATR/ATM Gene Interactions 

The ATR/ATM radiation-induced genes involved in DNA repair response in *Arabidopsis* are shown as hub-genes (red color) in the subnetwork modules in Figure 2, Figure 3, Figure 5 and Figure 6. We have identified other non-radiation-induced gene players from the DDR processes. These genes encode proteins that are involved in DNA repair as well as other responses to Reactive Oxygen Species (ROS) and oxidative stress, osmotic stress, salt stress, light stress, cell growth, and metabolic processes. The pigment biosynthesis process subnetworks shown in Figure 4 (LRD4—GLDS 120 dataset) reveal significant genetic interactions with non-pigment genes, as well. This dataset was collected from *Arabidopsis* grown for 11 days in the ambient light of ISS [22]. None of the flavonoids and carotenoids show significant regulations in the other low radiation dose datasets, and very high doses of HZE and γR radiation datasets, and the reason for this could be the absence of light stimulation.

In Figure 2, Figure 3, Figure 5 and Figure 6 the AT2G30250 (WRKY) transcription factors and other *Arabidopsis* ATR and ATM orthologues identified as hub genes are activated by several downstream target genes of stress signaling. The role of the ATR and ATM as the initiators of comprehensive signal transductions in response to DNA damage and/or ionizing radiation is probably achieved at the post-translational level [9,61]. Figure 4B represents the ABA biosynthetic activation as part of the stress responses which involves some of the carotenoid catabolic pathways.

The HZE and γR radiation DDR responses of the plant exhibit complex subnetworks for the DNA metabolic and the nucleic acid processes in response to very high doses of radiation. The genes AT3G13380, AT5G61600, AT5G48720, and AT3G27060 in low radiation dose subnetworks play key roles in the regulation of plant biological processes enabling the plant to be repair proficient, thereby inhibiting any phenotypic changes during growth. The causal associations of key ATR/ATM genes with other genes show the most relevant genes important for the adaptation of the plant to different levels of radiation. The 18 genes AT3G13380, AT3G61630, AT5G61600, AT3G27060, AT3G51920, AT1G70940, AT5G48720, AT2G31320, AT4G21070, AT2G46610, AT3G21280, AT1G07500, AT5G66140, AT1G27940, AT3G60420, AT4G28950, AT1G23000, and AT1G15580 identified in this research are essential regulators of the processes in response to DNA damage. Table 8 summarizes the functions of the ATR/ATM genes which shows clearly that these genes are not only involved in DNA damage response but are also involved in other biological processes. 

Table 9 summarizes the functions of the non-radiation-induced key genes encoding proteins that have significant regulatory activity and are also involved in non-DDR processes such as stress responses specifically to salt, water, heat, and light stress and in regulating plant development and root growth. The top five genes are the non-radiation-induced gene players identified from the DNA metabolic process subnetwork in Figure 3. The bottom 11 genes are identified from causal relational subnetworks. 

The ATR/ATM radiation-induced genes respond to DNA damage and/or ionizing radiation in concert with non-radiation-induced genes that respond to other environmental stressors such as ROS, salt, light, heat, and water changes in spaceflight. The MB causal relational subnetworks show the ATR/ATM genes related with genes that regulate root development, and plant growth, indicating that increased radiation tolerance by genetic activations may have a side effect on the plant growth. This fact has to be considered while genetically engineering plants for DNA repair response which may have undesired side-effects such as reduced yield. Further experiments are necessary to study the effect of long-term exposure of plants to space radiation and the best mechanisms to overcome the harmful effects of radiation. The overexpression of several genes and their side effects may be averted by suitable enclosures that can shield the plant from spaceflight radiation. 

## 5. Conclusions

We have applied advanced network analysis and machine learning approaches to detect key genes involved in DDR and their links with genes involved in spaceflight stress responses in *Arabidopsis*. Our analysis shows that the DDR processes are intermixed with the regulation of critical functions necessary for the plant to adapt to the spaceflight environment. There is a significant difference in the regulation of these processes in a low radiation dose environment where ATR/ATM radiation-induced genes interact with many downstream genes, whereas in the very high HZE and γR radiation dose environment, the DDR processes are regulated chiefly by ATR/ATM genes.

## Figures and Tables

**Table 1:** Network measures for hub genes in the subnetworks for the cellular response to stimulus process under low radiation dose.

*Arabidopsis* Gene Identifier for Hub Genes	Degree Distribution		Subgraph Centrality	
	LRD1	LRD2	LRD1	LRD2
AT1G08260	1	1	1.5431	1.5431
AT1G29440	1	1	1.5431	1.5431
AT2G30360	1	4	1.5431	1.5431
AT3G13380	12	7	15.9895	7.0825
AT3G51920	6	1	5.8344	1.5431
AT3G61630	2	13	2.1782	18.4146
AT4G28950	1	3	1.5431	2.9146
AT5G07100	1	9	1.5431	10.0677
AT5G40840	4	4	3.7622	3.7622
AT5G48720	1	20	1.5431	3.7622
AT5G61600	1	15	2.1782	18.4146

**Table 2:** Network measures for hub genes in the subnetworks for the cellular response to stimulus process under low radiation dose.

*Arabidopsis* Gene Identifier for Hub Genes	Degree Distribution		Subgraph Centrality	
	LRD3	LRD4	LRD3	LRD4
AT3G27060	2	22	2.3811	6253.2089
AT3G51920	4	40	4.9976	11,529.486
AT1G70940	1	22	1.5907	12,412.744

**Table 3:** Network measures for hub genes in the subnetworks for the cellular response to stress process under low radiation dose.

*Arabidopsis* Gene Identifier for Hub Genes	Degree Distribution		Subgraph Centrality	
	LRD1	LRD2	LRD1	LRD2
AT1G08260	1	1	1.5431	1.5431
AT5G40840	4	4	3.7622	3.7622
AT5G07100	1	9	1.5431	10.0677
AT5G48720	1	20	1.5431	43.7775

**Table 4:** Network measures for hub gene AT3G27060 in the subnetwork for the cellular response to stress process (row 1), cellular response to DNA damage stimulus (row 2) and DNA metabolic process (row 3) under low radiation doses.

DDR Processes	Degree Distribution		Subgraph Centrality	
	LRD3	LRD4	LRD3	LRD4
Cellular response to stress	3	22	54.4538	2.9737
Cellular response to DNA damage stimulus	2	33	2.1782	156.2451
DNA metabolic process	2	22	2.3811	57.2840

**Table 5:** Network measures for the subnetworks for low radiation dose DDR processes.

Cellular Response to Stimulus				
Network Measurements	LRD3	LRD4	LRD1	LRD2
Spectral gap	0.3347	6.172	2.013	0.099
Density	0.0489	0.0711	0.022	0.0037
Diameter	1	1	1	1
Conn. Comps.	3	2	3	28
Cellular Response to Stress				
Spectral gap	0.7923	4.69	0.99	0.099
Density	0.027	0.087	0.102	0.0104
Diameter	2	1	1	1
Conn. Comps.	9	1	5	17
DNA Metabolic Process				
Spectral gap	0.858	1.202	0.99	0.4112
Density	0.5714	0.038	0.166	0.0059
Diameter	1	1	1	2
Conn. Comps	6	2	3	69
Cellular Response to DNA Damage Stimulus				
Spectral gap	0.068	5.74	0.99	0.1399
Density	0.055	0.058	0.166	0.0074
Diameter	1	1	1	2
Conn. Comps.	7	1	3	55

**Table 6:** Network measures for the subnetworks for very high HZE and γR radiation dose DDR processes.

Network Measurements	DNA Metabolic Process		Nucleic Acid Response Process	
	HZE	γR	HZE	γR
Density	0.1782	0.2040	0.2465	0.2211
Spectral gap	7.5153	8.9726	12.2554	10.4919
Diameter	2	4	2	5

**Table 7:** Jaccard similarity index between subnetworks for different levels of radiation doses.

**Subnetwork with Hub-Gene**	**Jaccard Similarity Index between HZE and γR Subnetworks**	
	**DNA Metabolic Process**	**Nucleic Acid Response Process**
AT1G09815	0.5789	0.5789
AT2G31320	0.6176	0.6176
AT4G21070	0.7619	-
AT1G13330	0.4814	0.4285
AT4G29170	0.4444	0.4444
AT2G46610	-	0.6250
Subnetwork with hub-gene	Jaccard similarity index between LRD3 and LRD4 subnetworks	
	DNA metabolic process and Response to DNA damage to stimulus	DNA metabolic process & Response to DNA damage to stimulus
AT3G27060	1.0	0.1224

**Table 8:** Key ATR/ATM radiation-induced genes identified from the DDR process subnetworks.

*Arabidopsis* Gene Identifier	Gene Coding Protein Description
AT3G13380	Protein binding, protein serine kinase activity
AT3G61630	Cotyledon development, embryo development ending in seed dormancy, leaf development, regulation of transcription, DNA-templated
AT5G61600	Cell division, defense response to fungus, phloem or xylem histogenesis, positive regulation of transcription, DNA-templated
AT3G27060	Directly involved in synthesis of deoxyribonucleotides, DNA repair, DNA replication, multicellular organism development, programmed cell death, regulation of cell cycle
AT3G51920	Response to salt stress and water deprivation, calcium ion binding.
AT1G70940	Positive gravitropism, regulation of root meristem growth, response to light stimulus, root development, root hair elongation, root hair initiation
AT5G48720	DNA repair, female meiotic nuclear division, pollen development, response to X-ray
AT2G31320	DNA ADP-ribosylation, DNA repair, double-strand break repair, protein ADP-ribosylation, protein poly-ADP-ribosylation, response to abscisic acid, response to oxidative stress
AT4G21070	DNA recombination, DNA repair, cellular response to gamma radiation, double-strand break repair via homologous recombination, negative regulation of fatty acid biosynthetic process
AT2G46610	mRNA splicing, via spliceosome, RNA binding, protein binding
AT3G21280	Protein deubiquitination, ubiquitin-dependent protein catabolic process
AT1G07500	Cellular response to DNA damage stimulus, negative regulation of mitotic nuclear division, regulation of DNA endoreduplication
AT5G66140	Proteasomal ubiquitin-independent protein catabolic process
AT1G27940	ATPase-coupled transmembrane transporter activity and nucleotide binding
AT3G60420	Phosphoglycerate mutase family protein
AT4G28950	Meiotic DNA repair, pollen development, and responds to X-ray
AT1G23000	Heavy metal transport/detoxification superfamily protein involved in metal ion transport
AT1G15580	Regulation of transcription, DNA-templated, response to auxin

**Table 9:** Key non-radiation-induced genes interacting with ATR/ATM radiation induced genes.

*Arabidopsis* Gene Identifier	Gene Coding Protein Description
AT1G01470	Induced in response to wounding and light stress. Might be involved in protection against desiccation.
AT1G06390	This gene is involved in response to osmotic stress. This protein can interact with the BZR1 protein involved in brassinosteroid-mediated signaling in a Y2H assay and promotes BZR1 phosphorylation in protoplasts.
AT1G05850	Essential for tolerance to heat, salt and drought stresses. Also involved in root hair development, cell expansion and response to cytokinin.
AT1G05680	This enzyme can also transfer glycosyl groups to several compounds related to the explosive TNT when this synthetic compound is taken up from the environment.
AT1G05620	Transcript levels for this gene are elevated in older leaves suggesting that it may play a role in purine catabolism during senescence.
AT3G22370	Plays a role in shoot acclimation to low temperature. Also is capable of ameliorating reactive oxygen species production when the cytochrome pathway is inhibited.
AT5G43680	The protein is localized to the inner mitochondrial membrane that is nuclear-encoded and is essential for plant growth and development.
AT2G19620	Plays a role in dehydration stress response.
AT4G34410	Direct participation in auxin biosynthesis leading to the plant’s ability to tolerate salt stress.
AT2G19620	Plays a role in dehydration stress response.
AT4G31480	Required for plant growth, salt tolerance, and maintenance of the structure of the Golgi apparatus.
AT1G72490	It is expressed in roots and involved in leaf root architecture, specifically the orientation of lateral root angles
AT5G61020	Involved in cell proliferation during plant organogenesis.
AT5G45420	Plays a role in root hair elongation.
	Important role in controlling root skewing and maintaining the microtubule network.
AT4G35100	Salt-stress-inducible Major Intrinsic Protein (MIP)
AT1G13900	Encodes a dual-localized acid phosphatase (mitochondria and chloroplast) that modulates carbon metabolism.

## Data Availability

https://www.genelab.nasa.gov, accessed on 1 August 2020.

## Supplementary Materials

The following are available online at https://www.mdpi.com/article/10.3390/genes12060938/s1, Supplementary Table S1. Radiation Metadata for GLDS-7, GLDS-37, GLDS-38, and GLDS-46 datasets. GLDS-120 dataset was acquired under similar conditions of GLDS-7. Supplementary Table S2. Gene names for the ATG numbers for hub genes and authority genes in Figure 2, Figure 3 and Figure 4. Supplementary Table S3. Gene names for the ATG numbers for hub genes and authority genes in Figure 5 and Figure 6. The scripts of available at https://github.com/jjorozco777/Radiation-Network-Analysis, accessed on 10 June 2021.
