# Supplementary material for: Detection of Genes in Arabidopsis thaliana L. Responding to DNA Damage from Radiation and Other Stressors in Spaceflight
Source: Genes (Basel). 2021 Jun 19;12(6):938. doi: 10.3390/genes12060938 (PMC8234954; doi:10.3390/genes12060938)
Supplement: Supplementary file 1 [file genes-12-00938-s001.zip › genes-1228120-supplementary.pdf]

Supplementary Table S1. Radiation Metadata for GLDS-7, GLDS-37, and GLDS-38 datasets. GLDS-120 dataset was acquired under similar conditions of GLDS-7.

Average Absorbed Dose Rate (mGy-day): low-LET (or SAA)	Average Absorbed Dose Rate (mGy-day): high-LET (or GCR)	Cumulative Absorbed Dose (mGy): low-LET (or SAA)	Cumulative Absorbed Dose (mGy): high-LET (or GCR)	Mission	Accession	Experiment Duration (Days)	Binominal Name
0.11	0.16	1.46	2.02	STS-129	GLDS-7	12	Arabidopsis thaliana
0.15	0.12	1.93	1.58	STS-129	GLDS-7	12	Arabidopsis thaliana
0.11	0.15	1.23	1.69	STS-129	GLDS-7	12	Arabidopsis thaliana
0.13	0.14	2	2.16	STS-129	GLDS-7	14	Arabidopsis thaliana
0.07	0.13	0.58	1.06	SpaceX CRS-4	GLDS-37	8	Arabidopsis thaliana
0.06	0.13	0.17	0.38	SpaceX-5	GLDS-38	3	Arabidopsis thaliana
	7 Gy/min		100Gy	NSRL	GLDS-46	5	Arabidopsis thaliana
	2.5 Gy/min		30 Gy	NSRL	GLDs-46	5	Arabidopsis thaliana

Supplementary Table S2. Gene names for the ATG numbers for hub genes and authority genes in Figures 2, 3, and 4.

Figure 2		Figure 3		Figure 4 flavonoid		Figure 4 carotenoid	
**ATG number**	**Gene name**	**ATG number**	**Gene name**	**ATG number**	**Gene name**	**ATG number**	**Gene name**
AT2G45460	AT2G45460	AT1G02840	SR34	AT1G32640	MYC2	AT3G14440	NCED3
AT5G20850	RAD51	AT1G02850	BGLU11	AT1G03900	ATNAP4	AT1G02335	GL22
AT5G48720	XRI1	AT1G02870	AT1G02870	AT1G02850	BGLU11	AT1G06720	AT1G06720
AT1G73540	NUDT21	AT1G03110	TRM82	AT1G01470	LEA14	AT1G06900	AT1G06900
AT2G31320	PARP1	AT1G03230	AT1G03230	AT1G01720	NAC002	AT1G01470	LEA14
AT2G30250	WRKY25	AT1G03410	2A6	AT1G01800	AT1G01800	AT1G01720	NAC002
AT5G20930	TOUSLED	AT1G03860	PHB2	AT1G01820	PEX11C	AT1G01820	PEX11C
AT3G53280	CYP71B5	AT1G03870	FLA9	AT1G02305	CATHB2	AT1G02305	CATHB2
AT5G67360	SBT1.7	AT1G03910	CTN	AT1G04170	EIF2 GAMMA	AT1G03090	MCCA
AT5G67460	AT5G67460	AT1G04140	AT1G04140	AT1G05347	AT1G05347	AT1G03220	AT1G03220

AT2G06510	RPA1A	AT1G04170	EIF2 GAMMA	AT1G05575	AT1G05575	AT1G03610	AT1G03610
AT5G66130	RAD17	AT1G04183	AT1G04183	AT1G07135	AT1G07135	AT1G03850	AT1G03850
AT4G02390	PARP2	AT1G04190	TPR3	AT2G40890	CYP98A3	AT1G04440	CKL13
AT2G18760	CHR8	AT1G04270	RPS15A	AT1G06640	AT1G06640	AT1G05147	AT1G05147
AT5G65360	HTR2	AT1G04340	AT1G04340	AT1G02900	RALF1	AT1G05347	AT1G05347
AT5G40840	SYN2	AT1G04410	MDH1	AT1G02500	SAM1	AT1G06570	HPD
AT4G29170	MND1	AT1G04430	AT1G04430	AT1G06550	AT1G06550	AT1G07135	AT1G07135
AT5G64060	anac103	AT1G04440	CKL13	AT1G04870	PRMT10	AT1G07140	RANBP1A
AT4G19130	AT4G19130	AT1G04510	PRP19A	AT5G13930	CHS		
AT5G60250	AT5G60250	AT1G04680	AT1G04680	AT1G06110	SKIP16		
AT5G55490	GEX1	AT1G04690	KAB1	AT1G04250	IAA17		
AT5G52750	HIPP13	AT1G04750	VAMP721	AT1G06040	BBX24		
AT5G49480	ATCP1	AT1G04820	TUBA4	AT5G48930	HST		
AT5G49110	AT5G49110	AT1G04870	PRMT10	AT1G01210	AT1G01210		
AT5G47610	ATL79	AT1G04940	TIC20-I	AT1G06390	ASK9		
AT3G27060	TSO2	AT1G04980	PDIL2-2	AT1G03110	TRM82		
AT5G46740	UBP21	AT1G05260	PER3	AT1G04503	nan		
AT5G07100	WRKY26	AT1G05620	URH2	AT1G01550	BPS1		
AT5G43440	AT5G43440	AT1G05680	UGT74E2	AT1G02335	GL22		
AT5G24280	GMI1	AT1G05850	CTL1	AT1G05570	CALS1		
AT5G18270	ANAC087	AT1G05927	AT1G05927				
AT5G15380	DRM1	AT1G06040	BBX24				
AT5G15310	ATMYB16	AT1G06110	SKIP16				
AT5G12010	AT5G12010	AT1G06220	CLO				
AT5G11460	FLZ10	AT1G06390	ASK9				
AT5G03780	TRFL10	AT1G06515	AT1G06515				
AT5G02760	AT5G02760	AT1G06700	PTI11				
AT2G38340	DREB2E	AT1G07020	AT1G07020				
AT5G01970	AT5G01970	AT1G07080	AT1G07080				
AT3G60420	AT3G60420						
AT3G57550	AGK2						
AT3G51920	CML9						
AT3G47540	AT3G47540						
AT4G21070	BRCA1						
AT3G45730	AT3G45730						
AT4G34510	KCS17						
AT4G33160	AT4G33160						
AT4G31840	ENODL15						
AT4G29780	AT4G29780						

AT4G28950	ARAC7
AT4G27652	AT4G27652
AT4G27654	AT4G27654
AT4G27280	KRP1
AT4G25330	AT4G25330
AT4G25470	DREB1C
AT4G22610	AT4G22610
AT4G11740	PUX8
AT4G05370	nan
AT4G01450	AT4G01450
AT2G40610	EXPA8
AT1G66810	AT1G66810
AT1G78110	AT1G78110
AT3G25250	OXI1
AT3G17250	AT3G17250
AT3G10500	NAC053
AT1G69160	AT1G69160
AT1G27940	ABCB13
AT1G49050	APCB1
AT1G31280	AGO2
AT2G24850	TAT3
AT1G09180	ATSAR1
AT1G12020	AT1G12020
AT1G03440	AT1G03440
AT1G24150	ATFH4
AT2G23830	PVA31

Supplementary Table S3. Gene names for the ATG numbers for hub genes and authority genes in Figures 5 and 6.

**Figure 5**		**Figure 6**	
**ATG number**	**Gene name**	**ATG number**	**Gene name**
AT1G59660	NUP98B	AT5G20850	RAD51
AT1G17360	AT1G17360	AT5G40840	SYN2
AT1G74390	NEN3	AT5G02220	SMR4
AT1G27940	ABCB13	AT3G42860	AT3G42860
AT3G07800	TK1A	AT4G35740	RECQL3
AT3G14560	AT3G14560	AT4G29170	MND1
AT3G25250	OXI1	AT4G21070	BRCA1
AT3G27060	TSO2	AT1G13330	HOP2

AT3G20490	AT3G20490	AT2G31320	PARP1
AT3G12510	AT3G12510	AT2G46610	RS31A
AT4G02390	PARP2	AT2G38340	DREB2E
AT4G19130	AT4G19130	AT2G30250	WRKY25
AT4G22960	AT4G22960	AT5G67460	AT5G67460
AT4G25330	AT4G25330	AT1G09815	POLD4
AT4G28950	ARAC7	AT5G66140	PAD2
AT4G37490	CYCB1-1	AT5G64060	anac103
AT3G45730	AT3G45730	AT2G21790	RNR1
AT5G03780	TRFL10	AT5G55490	GEX1
AT5G11460	FLZ10	AT5G48720	XRI1
AT5G23910	AT5G23910	AT5G48020	AT5G48020
AT5G24280	GMI1	AT5G24280	GMI1
AT5G48020	AT5G48020	AT5G23910	AT5G23910
AT5G48720	XRI1	AT5G11460	FLZ10
AT5G55490	GEX1	AT1G07500	SMR5
AT5G60250	AT5G60250	AT5G03780	TRFL10
AT5G64060	anac103	AT3G45730	AT3G45730
AT1G13330	HOP2	AT4G37490	CYCB1-1
AT5G66140	PAD2	AT4G34510	KCS17
AT5G67460	AT5G67460	AT4G28950	ARAC7
AT2G21790	RNR1	AT4G25330	AT4G25330
AT1G07500	SMR5	AT4G19130	AT4G19130
AT5G02220	SMR4	AT4G02390	PARP2
AT4G29170	MND1	AT1G20350	TIM17-1
AT4G25580	AT4G25580	AT3G12510	AT3G12510
AT1G09815	POLD4	AT3G20490	AT3G20490
AT2G31320	PARP1	AT3G27060	TSO2
AT4G21070	BRCA1	AT3G14560	AT3G14560
AT4G35740	RECQL3	AT3G07800	TK1A
AT3G42860	AT3G42860	AT1G74390	NEN3
AT5G40840	SYN2	AT1G17460	TRFL3
AT5G66130	RAD17	AT1G20180	AT1G20180
AT5G20850	RAD51	AT1G31280	AGO2
AT2G45460	AT2G45460	AT2G18600	RCE2
		AT2G47680	AT2G47680
